# Deciphering the mutation spectrum in south Indian children with congenital anomalies of the kidney and urinary tract

**DOI:** 10.1186/s12882-021-02628-z

**Published:** 2022-01-03

**Authors:** Ambili Narikot, Varsha Chhotusing Pardeshi, A. M. Shubha, Arpana Iyengar, Anil Vasudevan

**Affiliations:** 1 Divsion of Molecular Medicine, St. John’s Research Institute, St. John’s Medical College, Bengaluru, India; 2 Department of Pediatric Surgery, St. John’s Medical College, Bengaluru, India; 3 Department of Pediatric Nephrology, St. John’s Medical College, Bengaluru, 560034 India

**Keywords:** CAKUT, Genetics, Next generation sequencing, Variants, Monogenic

## Abstract

**Background:**

Congenital anomalies of the kidney and urinary tract (CAKUT) cover a spectrum of structural malformations that result from aberrant morphogenesis of kidney and urinary tract. It is the most prevalent cause of kidney failure in children. Hence, it is important from a clinical perspective to unravel the molecular etiology of kidney and urinary tract malformations. Causal variants in genes that direct various stages of development of kidney and urinary tract in fetal life have been identified in 5–20% of CAKUT patients from Western countries. Recent advances in next generation sequencing technology and decreasing cost offer the opportunity to characterize the genetic profile of CAKUT in Indian population and facilitate integration of genetic diagnostics in care of children with CAKUT.

**Methods:**

Customized targeted panel sequencing was performed to identify pathogenic variants in 31 genes known to cause human CAKUT in 69 south Indian children with CAKUT. The NGS data was filtered using standardized pipeline and the variants were classified using ACMG criteria. Genotype and phenotype correlations were performed.

**Results:**

The cohort consisted of children mostly with posterior urethral valve (PUV) (39.1%), vesico-ureteric reflux (VUR) (33.3%) and multi-cystic dysplastic kidney (MCDK) (7.2%). No pathogenic or likely pathogenic variants were identified in the study. Most of our variants (*n* = 39, 60%) were variants of unknown significance with 25.6% (10/39) of them were identified as potentially damaging but were novel variants.

**Conclusions:**

The present study did not identify any disease-causing monogenic variants in the cohort. The absence of genetic cause may be due to limitations of panel-based testing and also due to higher proportion of children with abnormalities in lower urinary tract than hypodysplasia of kidneys. Clinical, larger targeted panel or whole exome sequencing may be a better method to characterize the genetic profile of Indians patients with CAKUT.

**Supplementary Information:**

The online version contains supplementary material available at 10.1186/s12882-021-02628-z.

## Introduction

Congenital anomalies of the kidney and urinary tract (CAKUT) comprises of a spectrum of defects involving kidney (hypoplasia and dysplasia), ureters and bladder (vesico-ureteral reflux, duplex system) and urethra (posterior urethral valves) that results from perturbations in the development of the kidney and urinary tract during fetal life [1, 2]. The malformations may affect the kidney and urinary tract unilaterally and bilaterally or may occur in syndromic forms with other organ defects [3]. CAKUT occurs in about 5 per 1000 live births and contributes to 40–50% of end-stage kidney disease (ESKD) in children worldwide [4, 5].

Evidence from animal models, familial clustering and the occurrence of CAKUT in syndromic forms are indicative of genetic basis of the disease [6]. More than 50 single gene defects have been identified in 10 to 12% of isolated CAKUT [7]. These are inherited in recessive or dominant form with incomplete penetrance and variable expressivity, resulting in genetic heterogeneity and complex inheritance of CAKUT. Detection of a causal variant in children with CAKUT is important for confirming the molecular cause, to facilitate early recognition of extra renal abnormalities and prompt treatment of clinically significant co-morbidities. In view of lack of genetic screening studies in Indian population, information about genes and the frequency of variants in these genes in Indian children with CAKUT is unknown which is a major barrier to the routine use of genetic testing in the clinic.

Significant advances in next-generation sequencing based genetic testing have immensely enhanced knowledge of genetic basis of many diseases and improved patient care and genetic counseling. Apart from cost-effectiveness, heterogeneity in CAKUT with multiple genes being implicated with diverse modes of inheritance along with significant phenotypic variability justifies the use of next-generation sequencing (NGS) as a tool for genetic screening [7]. In conditions associated with multiple single gene defects, gene panel-based NGS is a cost effective method for the identification of casual variants in a specific set of disease associated genes and also prevents the identification of incidental variants [8–10]. However, selection of genes for the NGS panel and diagnostic yield has not been evaluated in most of the ethnic backgrounds, including in Indian population.

The aim of the present study was to determine the utility of NGS based customized targeted gene panel for genetic diagnosis of CAKUT and to know the genetic spectrum in south Indian children with CAKUT. We screened common CAKUT phenotypes in children using customized NGS based gene panel.

## Materials and methods

### Patients and clinical data

In this prospective study, children diagnosed with CAKUT (age group: newborn − 18 years) were enrolled for the study from pediatric nephrology and surgery units between Jan 2016 and Jan 2019. Both incident and prevalent cases with follow up for at least 6 months were considered for recruitment. Institutional Ethics Committee approved the study (IEC Ref No- 163/2016) and all participants were recruited after informed consent. The diagnosis of CAKUT was made by pediatric nephrologists or surgeon on the basis of the following: ultrasonography (USG) of kidney and urinary tract, DMSA (Dimercaptosuccinic acid) scan and Micturating Cysto-urethrogram (MCUG), to identify any abnormality of number, size, shape, or anatomic position of the kidneys or other parts of the urinary tract. It included participants who had at least one of the following: vesicoureteral reflux (VUR) with or without hypodysplasia, posterior urethral valve (PUV), uretero–pelvic junction obstruction (UPJO), vesico-ureteric junction obstruction (VUJO), multicystic dysplastic kidney (MCDK), isolated renal hypodysplasia which is characterized by small kidney size with altered echogenicity, unilateral renal agenesis, duplex collecting system, megaureter and horseshoe kidney [6, 11]. Both, syndromic (defined as CAKUT with extra renal features like optic nerve coloboma, ear fistula, pinna deformation, hearing loss, cardiac, gastrointestinal, neurological or skeletal defects) and non-syndromic cases (defined as CAKUT without extra renal features) were included in the study. Patients with a known causative genetic or chromosomal abnormality, isolated neurogenic bladder, and those who have undergone nephrectomy for medical reasons such as Wilm’s tumor, refractory hypertension were excluded from the study. Clinical data, laboratory and imaging details were documented. Glomerular filtration rate (GFR) was estimated using revised Schwartz equation. Hypertension, chronic kidney disease (CKD) and ESKD was defined as per standard criteria [12]. For children less than 2 years, age-specific normal ranges for GFR was used to diagnose CKD [13].

### Next generation sequencing

Blood sample (EDTA 5 ml) collected from patients at the time of recruitment was further processed for extraction of DNA. Genomic DNA was extracted from leukocytes using QIAamp blood mini kit (Qiagen, Hilden, Germany) as per manufacturer’s instructions. Quantity of extracted DNA was estimated using Qubit fluorometric assay (Thermofisher scientific, MA, USA).

Genes for the customized panel were selected through a literature search in various online public databases such as OMIM (Online Mendelian Inheritance in Man) database (http://omim.org/) and PUBMED (http://www.ncbi.nlm.nih.gov/pubmed/) (Dec 2015). The following keywords were used: CAKUT, urinary tract anomalies or abnormalities, multicystic kidney dysplasia, vesico-ureteral reflux, duplex collecting system, posterior urethral valve, uretero–pelvic junction obstruction and renal/kidney diseases, genetic testing and mutations in CAKUT. Thirty-one genes associated with CAKUT were finally selected after a review of published studies in different ethnicities. Customized gene panel consisting of 31 genes was designed using Ion-Ampliseq primer designer (Life Technologies), targeting the coding regions of the genes (Additional file 1.docx- Genes included in the customized CAKUT NGS panel). The panel consisted of 825 primers (3 primer pools) with amplicon size ranging from 125 to 375 bp, targeting the exonic regions (221.38 kb, 449 exons) with coverage of 98.99%. The uncovered regions were mainly repeat rich region making primer designing difficult.

Sixty-nine patients (3 syndromic children) with complete clinical and follow up data and representing various CAKUT phenotypes were selected for sequencing. The barcoded libraries were prepared using 20 ng DNA and HQ-Ion AmpliSeq Library Kit 2.0 (Thermo Fisher Scientific Inc. USA) as per the manufacturer’s instructions. Library quantity and quality was determined using Qubit fluorometric assay and Agilent BioAnalyzer High-Sensitivity DNA kit (Agilent Technologies, CA, USA), respectively. The libraries were amplified using Ion-Ampliseq Hi-Fi PCR mix, partially digested and phosphorylated and barcoded using Unique-Ion express barcodes. The barcoded libraries were pooled and enriched using Ion PGM Template OT2 400 kit (Thermo Fisher Scientific Inc. USA) according to the manufacturer’s protocol. Next generation sequencing was carried out using the Ion PGM Hi-Q Sequencing Kit (318 Chip, Thermo Fisher Scientific Inc. USA) on Ion Torrent Personal Genome Machine sequencer (Thermo Fisher Scientific Inc. USA) as per the manufacturer’s protocol.

### NGS data analysis

Analysis was carried out using Ion Torrent Suite™ Browser version 5.0 and Ion Reporter™ version 5.0. Torrent Suite™ Browser was used to perform initial quality control including chip loading density, base calling, alignment (hg19/GRCh37), median read length and number of mapped reads, assembly, coverage analysis and variant calling. Variants were identified by Ion Reporter using in house developed and validated filtering criteria: variant coverage >20x,variant type and effect (non-synonymous, frame-shift, nonsense), location (to detect splice site variants) and variants with a minor allele frequency (MAF) < 1% in public databases (Gnomad (https://gnomad.broadinstitute.org/), ExAc and 5000 Exome (http://evs.gs.washington.edu/EVS/) [14]. All frame-shift variants and variants affecting stop codons were retained irrespective of their MAF. Synonymous variants except those located ±2 bp off exon boundaries and intronic variants > 10 bp from exon boundaries were removed. Variants with a minor allele frequency (MAF) higher than 0.01 in online databases ExAC and 1000 genome, were excluded. The filtered variants were manually inspected with a high-performance visualization tool - Integrative Genomics Viewer (IGV) ( [15]) to filter out variants with possible strand-bias and variants within homopolymeric region. The functional significance of the filtered variants were evaluated *in-silico* using online prediction software SIFT [16], Polyphen-2 ( [17]) and MutationTaster [18]. Variants of interest were classified as pathogenic, likely pathogenic, variants of uncertain significance (VUS), likely benign, or benign using American College of Medical Genetics and Genomics (ACMG) guidelines and Sherloc guidelines by Nykamp et.al, which was a refinement of ACMG guidelines [19, 20]. A scoring system developed by Karbassi et al. was used to determine the pathogenicity of VUS [21].

## Results

### Patient characteristics

**Fig. 1:**
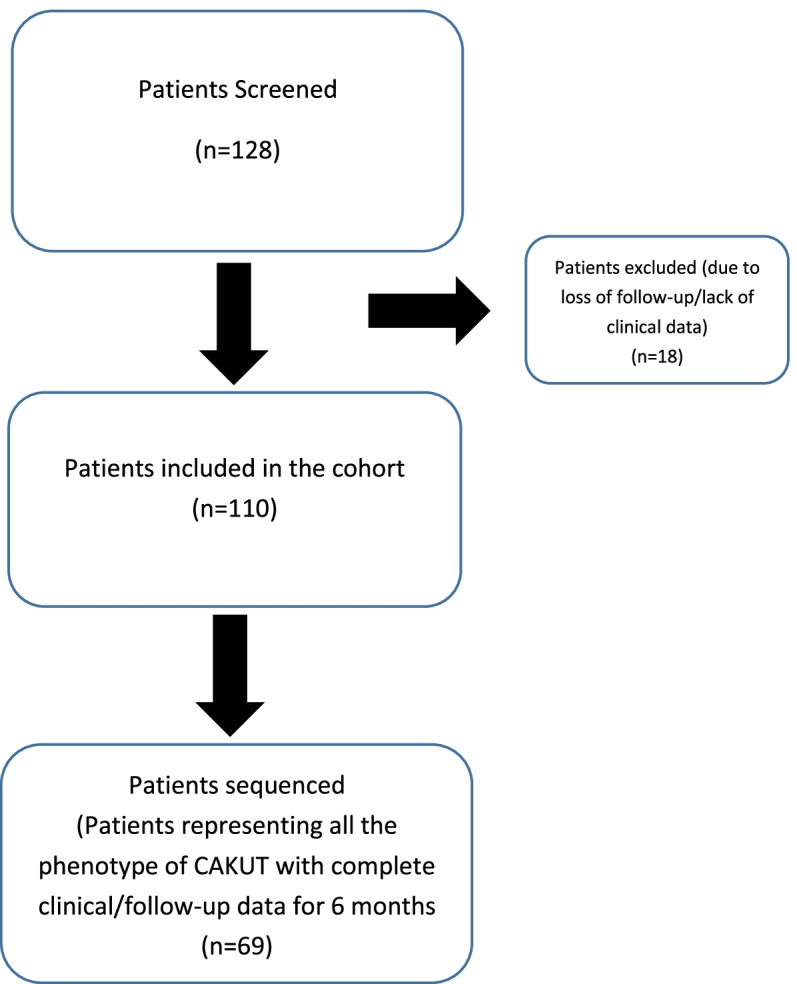
Flowchart of the children with congenital anomalies of the kidney and urinary tract cohort development

A flow diagram of recruitment process for CAKUT is shown in Fig. 1. A total of 128 patients were screened and 110 children diagnosed with CAKUT were included in the study. Eighteen children with no follow up data or without a conclusive diagnosis were excluded. All 110 patients could not be included for sequencing due to cost constraints. Patients with complete clinical data and follow up data of at least 6 months were selected for sequencing. Also, proportionate weightage was given to each diagnosis in entire cohort (Table 1). The clinical characteristics of patients included in the whole cohort (*n* = 110) as well as those sequenced (*n* = 69) is given in Table 1. PUV and VUR were most common diagnosis observed. In the sequenced cohort, 27 subjects had abnormalities of kidneys and urinary tract suggestive of CAKUT in antenatal ultrasound. In the rest CAKUT was diagnosed after birth, with median age at diagnosis of 1 year (IQR- 0.75 - 3) (Additional file 2: Table S1.xlsx -Detailed clinical profile of cohort included in the study). The median age at recruitment was 6 years (IQR- 4.5-9). The most common diagnosis was VUR (*n* = 42) and PUV (*n* = 37) (Table 1). History of consanguinity was observed in 15 (21.73%) patients. Three patients had extra renal manifestations which included anorectal malformations (ARM), recto vestibular fistula, congenital heart defect (CHD), atrial septal defect (ASD) and urogenital sinus. Of the 69 subjects sequenced, 9 patients (13.04%) exhibited impaired renal function (defined as CKD ≥ stage 2with estimated glomerular filtration rate ≤ 60 mL/min/1.73 m2) at the recruitment, and 3 of them (4.34%) progressed to CKD at the median age of 12 years (IQR- 6-12) (Table 1).

**Table 1:** Clinical characteristics of South Indian Children with congenital anomalies of the kidney and urinary tract

Patient Characteristic	Study cohort (n = 110)	Sequenced Cohort (n = 69)	*p* value*
Age (yrs.) (median;IQR)	5 (5–9.2)	6 (4.5–9)	
Gender (Male: Female)	96:14	56: 13	
Renal Phenotypes:			
1. Vesico-Ureteric Reflux (VUR) (Bilateral; Unilateral)	42 (20:22) (38.18%)	23(14:9) (33.33%)	0.52
a. VUR with Hypodysplasia	26 (23.63%)	10 (14.49%)	
b. VUR without Hypodysplasia	16 (14.54%)	13 (18.84%)	
2. Hypodysplastic kidney	4 (3.63%)	0	0.30
3. Posterior Urethral Valve (PUV)	37 (33.63%)	27 (39.13%)	0.45
4. Duplex Collecting System	4 (3.63%)	3 (4.34%)	1.00
5.Multi-Cystic Dysplastic Kidney (MCDK)	7 (6.36%)	5 (7.24%)	1.00
6. Ureteropelvic Junction Obstruction (UPJO) (Bilateral: Unilateral)	5 (1:4, 4.54%)	5 (1:4, 7.24%)	0.51
7. Vesicoureteral Junction Obstruction (VUJO) (Bilateral: Unilateral)	2 (0:2, 1.81%)	2 (0:2, 2.89%)	0.64
8. Renal Agenesis	7 (6.36%)	3 (4.34%)	0.74
9. Horseshoe Kidney	1 (0.9%)	1(1.44%)	1.00
10. Ectopic kidney	1 (0.9%)	0	1.00
Family history	2 (1.81%)	0	
Consanguinity^a^	27 (24.54%)	15 (21.73%)	
Syndromic	8(7.27%)	3 (4.34%)	
No of subjects with CKD (≥ stage 2) @ recruitment	21 (19.09%)	9 (13.04%)	

^a^As reported by parents *chisquare test between diagnostic category in study cohort and sequenced cohort

### Genetic analysis

Five sequencing runs to screen 69 subjects generated 1.57 GB of Q20 data. The coverage was comparable between runs (Additional file 2: Table S2.xlsx -Ion PGM next-Generation Sequencing run summary). Combining the data derived from five runs, sequencing generated a mean of 0.19 M reads per individual with mean read length of 229 bp. On an average, 99.2% of these reads mapped to the reference genome and 97% were on target. Mean coverage of 190× was achieved for the genes across all samples, with 86, 53 and 9% of the targets having minimum read depth of 20×, 100× and 500× respectively (Additional file 2: Table S3.xlsx -Summary of per sample NGS data output and quality in Indian Congenital Anomalies of Kidney and Urinary Tract cohort).

**Fig. 2:**
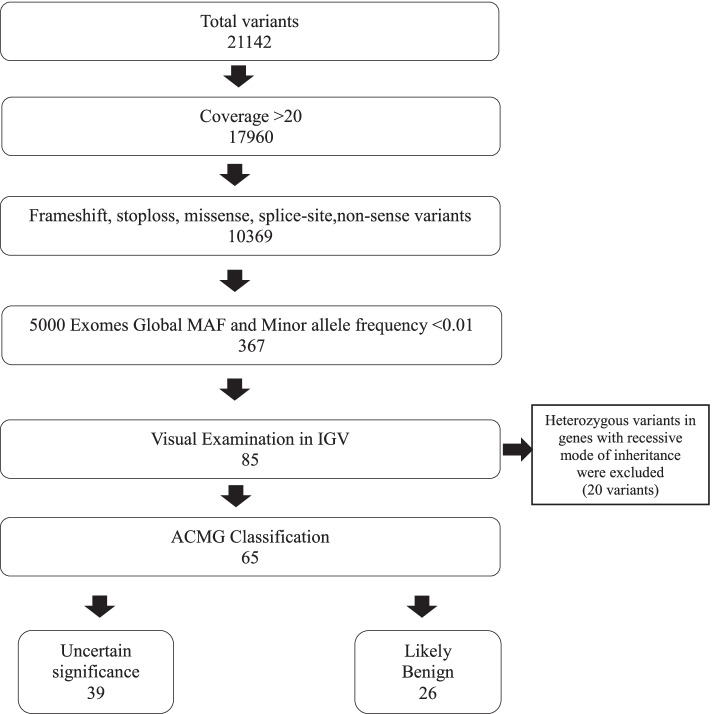
Schematic visualization of the variant filtration workflow used in this study

A total of 21,142 single-nucleotide variants (SNVs) and indels were obtained and these variants were annotated and filtered using Ion Reporter software 5.1 with parameters as given in Fig. 2. Following filtration, 367 variants were obtained which were reviewed manually, by examining quality scores and visually inspecting the data in Integrative Genome Viewer (IGV; Broad Institute). A total of 282 variants were removed subsequently due to strand bias, poor mapping in IGV, presence of variant in homopolymeric region and not well sequenced clean neighborhood. Following IGV, 85 variants were obtained, and from these 20 heterozygous variants identified in genes with recessive mode of inheritance were excluded. The remaining 65 variants were classified according to standard ACMG criteria; 39 variants of unknown significance (60%) and 26 likely benign variants (40%) were identified. Fourteen variants out of 65 variants (21.5%) were novel. The lists of variants identified in each patient are listed in Additional file 2: Table S4.xlsx (List of per patient variant identified in the cohort).

### Genotype-phenotype correlation

Majority (60%) of identified variants were of unknown significance of which 14 variants were novel i.e. absent in population databases. Even though no pathogenic or likely pathogenic variants were identified in the study, 10 variants in 5 genes were identified as potentially damaging based on scoring criteria (*CHD1L* (*n* = 1), *FRAS1* (*n* = 3), *TNXB* (*n* = 4), *FREM2* (n = 1), *SIX5* (n = 1)) (Additional file 2: Table S5.xlsx -Pathogenicity risk score calculation for Variant of unknown significance). The phenotypes observed in patients with these 7 variants were UPJO, duplex collecting system and renal agenesis for *FRAS1* variants, VUR, L-Duplex ureter, PUV and horse-shoe kidney (syndromic) for *TNXB* variants, VUR for *FREM2* variant, PUV for *CHD1L* gene and VUJO with Duplex system for variant in *SIX5* gene. Patient CT-51, with PUV had variants in two genes, [*TNXB* (p. Gln286fs), and *CHD1L* (p. Ser837fs)]. Another patient (CT-71) with similar *TNXB* (p. Gln286fs) gene variant had left duplex system with VUR in the lower moiety of left kidney. Two variants were identified in the same gene (*TNXB*) in two patients with different malformations, but the type of variants reported were different. The child with PUV (CT-51) had a deletion (c.857delA) while an insertion (c.856_857insG) was identified at the same chromosome position (chr6:32064772) in child with duplex system (CT-71). Although three variants [*TNXB* (p. Gln286fs), *CHD1L* (p. Ser837fs) and *TNXB* (p. Gln286fs)] are loss-of-function variants and shown to be damaging using computational tools, they were classified as VUS because they are not constrained against Loss-of-function (pli = 0). Three novel missense variants which were absent in Gnomad were identified in *PAX2* (p.Ser305Leu in child with unilateral hypodysplastic kidney), *SALL1* (p.Phe447Tyr in patient with bilateral VUR) and *RET* genes (p.Gly533Ser in child with unilateral vesico-ureteric junction obstruction).

## Discussion

Identifying genetic etiology of CAKUT poses a unique challenge due to clinical variability and genetic heterogeneity. Targeted re-sequencing approach has shown great potential in simultaneous testing of large numbers of patients and multiple known disease causing genes in a various renal disorders [22–24]. Therefore, a customized gene panel of CAKUT was developed and tested for its utility in genetic diagnosis and to determine genetic profile of CAKUT in Indian children.

The present study (*n* = 69) included isolated and syndromic CAKUT cases representing the full spectrum of CAKUT phenotypes. Although, no pathogenic or likely pathogenic variants were identified, it is important to report VUS as these variants could be reclassified as pathogenic or likely pathogenic if additional patients with same phenotype and same variants are reported in future. After determining the pathogenic score of the VUS variants using Karbassiet.al. Scoring system, 10 VUS variants which are potentially damaging were identified in 5 genes (*FRAS1, TNXB*, *FREM2*, *SIX5 and CHD1L*). Interestingly two cases with different CAKUT phenotypes (case 51 with PUV and case 71 with L Duplex Ureter system) showed single gene variant (*TNXB*) gene at the same position although the variants reported were different (c.857delA and c.856_857insG respectively). Previous Genome Wide Linkage Study (GWLS) and Whole Exome Sequencing (WES) study in a large kindred with VUR identified heterozygous pathogenic variants in *TNXB* as a cause of dominantly inherited VUR and joint hypermobility [25]. In the present study variant in *TNXB* gene was observed in duplex ureter system phenotype with reflux in lower moiety. The patient with *CHD1L* variant showed a similar phenotype of PUV that has been reported previously in a study conducted by Hwang et al. Authors identified variant in *CHD1L* gene in five unrelated families (0.76%) out of 650 families of diverse ethnic background. The phenotypes of individuals carrying the *CHD1L* gene variant is very heterogenous: bilateral kidney malrotation, right renal dysplasia, horseshoe kidneys, right duplex collecting system, right MCDK, left UVJO and PUV [26]. Variants in two different genes may be due to the fact that kidney development is complex involving the interaction of distinct pathways. It is likely that defects in different genes involved in these pathways may result in similar phenotypes [11, 27–31]. These two variants identified in *CHD1L* and *TNXB* met most of the criteria of pathogenicity but due to lack of data for the loss of function, these variants were further classified as variants of unknown significance. The findings in the current study supports the observations from previous studies that phenotypic classification alone may not be useful to predict the primary genetic defect in CAKUT [32]. For *TNXB* and *FRAS1* gene variants, the observations were similar to that of previous studies; except for PUJO (*FRAS1*) and Horse-shoe kidney (*TNXB*), which were observed only in our cohort [25, 26, 33–35]. Three novel missense variants were identified in *PAX2, RET* and *SALL1* gene. Variant in *PAX2* [p.Ser305Leu] was identified in a child (CT 14) with bilateral VUR and unilateral hypo dysplastic kidney. *PAX2* belonging to the GDNF-RET signaling pathway plays a major role in morphogenesis and development of kidney and urinary tract [36]. *PAX2* related disorder have a variable clinical presentation with renal and ophthalmological abnormalities (60–70%), and sometimes with other abnormalities like hearing loss. On screening, child with the novel missense variant had no abnormality in the eye. In a Japanese cohort, of the seven patients with deleterious variants in *PAX2* gene who progressed to renal failure in childhood, six had truncating variants [37]. Like *PAX2*, *SALL1* also belongs to GDNF-RET signaling pathway. A missense variant (p.Phe447Tyr) was identified in a child (CT 88) with bilateral VUR. Although heterozygous variants of *SALL1* cause Townes-Brocks syndrome, comprising of facial dysmorphism, limb defects, kidney and urinary tract abnormalities and anorectal malformation, isolated kidney and urinary tract abnormalities have also been reported [38]. Targeted sequencing of 7 known CAKUT-causing dominant genes in 749 individuals identified novel missense variants in *SALL1* in 6 patients, of which 4 had unilateral or bilateral VUR [26]. In the same study, deleterious variants in *RET* gene was identified in three families with different abnormalities of kidney and urinary tract suggesting phenotypic heterogeneity. In this study, *RET* variant (p.Gly533Ser) was identified in child (CT 73) with unilateral vesico-ureteric junction obstruction. Nevertheless, information about frequency of *RET* as one of the gene associated with CAKUT is conflicting [39, 40].

The diagnostic yield using targeted panel sequencing in CAKUT is variable with yield not impacted by number of genes included in the panel. Some studies reporting low diagnostic range (2–15%) [1, 26, 41], while in few other studies, pathogenic variants were identified in 6–20% of patients with CAKUT by analyzing 5–30 genes [11, 34, 35, 42–45].Study in a Dutch population also reported low diagnostic rate (3%), where they sequenced 208 candidate genes in 453 subjects representing the full spectrum of CAKUT phenotypes [44]. However in another cohort, a higher molecular diagnosis rate (18%) was reported in a panel sequencing study of 330 candidate genes in a cohort of 204 unrelated CAKUT patients [43]. In the current study many variants were classified as VUS, mainly as they were novel, lacking functional evidence, and also due to lack of evidence for segregation with the disease. In addition, a major barrier to variant calling is the absence of database specific to Indian population for allele frequencies. Larger multi-center study will also help in identifying common variants in children with CAKUT.

These findings indicate that the major limitation in variant classification is absence of functional data and genotype data from diverse ethnic background. With increasing NGS sequencing in clinical setting, it is likely that these VUS may be later classified as pathogenic or likely pathogenic. Previous studies reported *PAX2* and *HNF1B* as the most frequently mutated genes in the European and American population with CAKUT. These studies predominantly consisted of children with renal hypodysplasia (RHD) [46–50]. This is in contrast to the present study, wherein children were non-syndromic (92%) with lower urinary tract obstruction phenotypes -VUR (33.3%) and PUV (39.1%). *HNF1B* pathogenic variants are more frequently associated with hypo dysplasia and cystic kidneys and are rare in isolated lower urinary tract defects such as PUV and VUR, which were the most common diagnosis in the present study [46, 50]. Hence it is not surprising that we did not find any deleterious variants in *PAX2* or *HNF1B*.

The difference in the diagnostic yield in current study when compared to other studies could be attributed due to the approach using targeted panel as well as due to differences in cohort size, type of CAKUT that constituted the cohort and, the family history of patients, and also the genes included for screening. For example, high diagnostic yield was seen in cohort of severe CAKUT phenotypes, syndromic cases, patients from consanguineous families and familial cases [51, 52].This also addresses the need for familial testing as it helps in classification of variant into pathogenic or benign. But due to the unavailability of the samples, sequencing or testing of parents were not performed. It is possible that the deleterious variants in genes in targeted panel are rare in Indian population (less than 1% of observed cases with CAKUT) or larger cohorts screening is essential to confirm the observation. Besides monogenic cause, three large cohort studies identified that 4.5–16.6% of patients with CAKUT carry pathogenic CNV [9, 10, 22, 23, 42, 53–55] while targeted panel can identify single exon CNVs, multi exon CNVs are better identified using Whole Exome/genome sequencing panels. It is likely that CNVs in gene(s) or pathogenic variants in the noncoding regions not targeted by our customized panel could be responsible for the disease in the cohort. It is also known that epigenetic factors and non-genetic environmental factors also contribute to the occurrence of CAKUT [56]. This underlines that yet to be uncovered genetic complexity and environmental factors may be involved in the development of the disease. Hence, using other genetic testing approaches like microarray and clinical or whole exome sequencing may help decipher the genetic spectrum of CAKUT. WES studies reported diagnostic yield of 11–14% in CAKUT patients and identified novel gene variants [1, 56–60] which is higher than that obtained with targeted panel [8, 53–55]. Exome sequencing have distinct advantages compared to targeted panel since it covers a larger number of genes as well identify other types of variants like CNVs but is more expensive compared to targeted sequencing. Targeted panels are phenotype-driven panels, featuring few hundred genes associated with kidney diseases. Its use in CAKUT may be limited due to significant phenotypic heterogeneity of CAKUT resulting in exclusion of genes associated with CAKUT of particular phenotype. It will be more useful to use exome sequencing based approach as many NGS-based targeted gene sets are built on an exome backbone. This allows the laboratory to use the same process for library preparation and sequencing leading to lesser cost. Such an approach not only facilitates targeted gene analysis but also in searching variants in remaining genes of clinical exome if targeted gene analysis fails to identify causal variants The low yield obtained using a targeted panel in this study and the need to frequently update the targeted panel for the newer discovered/novel CAKUT associated genes makes clinical exome sequencing a more favorable approach.

## Conclusion

This is the first genetic analysis study conducted in Indian children with CAKUT using customized targeted panel sequencing. Most of our identified variants were of unknown significance, out of which 10 variants needs further exploration to determine pathogenicity. Using an exome sequencing approach and analyzing for CNV may help in identifying more children with CAKUT with a genetic cause and it can also help improve our understanding of genetic fingerprint in CAKUT.

## Supplementary Information

**Additional file 1.**

**Additional file 2.**

## Data Availability

The datasets generated and/or analysed during the current study are available in the GitHub repository [https://github.com/RenalGeneticsLab-SJRI/CAKUT].

## Abbreviations

CAKUT: Congenital Anomalies of Kidney And Urinary Tract
NGS: Next-Generation Sequencing
USG: Ultrasonography
DMSA: Dimercapto succinic acid scan
MCUG: MicturatingCysto-Urethrogram
VUR: Vesico Ureteral Reflux
PUV: Posterior Urethral Valve
UPJO: Uretero Pelvic Junction Obstruction
VUJO: Vesico-Ureteric Junction Obstruction
MCDK: MultiCystic Dysplastic Kidney
RHD: Renal hypodysplasia
ARM: Anorectal Malformations
CHD: Congenital Heart Defect
ASD: Atrial Septal Defect
CKD: Chronic Kidney Disease
IGV: Integrative Genomics Viewer
VUS: Variants of uncertain significance
ACMG: American College of Medical Genetics and Genomics
IQR: Inter Quartile Range
CNV: Copy Number Variation
WES: Whole Exome Sequencing
GWLS: Genome Wide Linkage Study
