# Supplementary material for: Deciphering the mutation spectrum in south Indian children with congenital anomalies of the kidney and urinary tract
Source: BMC Nephrol. 2022 Jan 3;23:1. doi: 10.1186/s12882-021-02628-z (PMC8722277; doi:10.1186/s12882-021-02628-z)
Supplement: Supplementary file 1 — Additional file 1. [file 12882_2021_2628_MOESM1_ESM.docx]

**Additional file 1: Table 1.** Genes included in the customized CAKUT NGS panel

| **Sr No** | **Gene symbol** | **Gene**  **name** | **No. of exons** | **Gene**  **size**  **(bp)** | **Inheritance** | **Renal Phenotype/Disease/Syndrome** | **Extra-Renal Phenotype/Disease/Syndrome** |
| --- | --- | --- | --- | --- | --- | --- | --- |
| 1 | *ACE* | angiotensin converting enzyme | 25 | 4959 | Autosomal Recessive | Absence or incomplete differentiation of proximal tubules | Pulmonary hypoplasia (Potter sequence), skull abnormalities |
| 2 | *AGT* | angiotensinogen | 5 | 2291 | Autosomal Recessive | Reduced number of proximal tubules, short proximal tubules without brush border, atrophic loops of Henle and collecting ducts, closely packed glomeruli, marked thickening and disorganization of interlobular and preglomerular arteries | Similar to ACE |
| 3 | *AGTR1* | angiotensin II receptor, type 1 | 2 | 2359 | Autosomal Recessive | Like AGT phenotype, PUV | Like ACE |
| 4 | *BMP4* | bone morphogenetic protein 4 | 4 | 1917 | Autosomal Dominant | Renal hypodysplasia | Cleft lip, microphthalmia |
| 5 | *CHD1L* | Chromodomain Helicase DNA Binding Protein 1-Like; | 23 | 2967 | Autosomal Dominant | hypodysplastic kidney/ hydronephrosis obstructive megaureters, PUV | - |
| 6 | *EYA1* | EYA Transcriptional Coactivator And Phosphatase 1 | 18 | 4326 | Autosomal Dominant | Unilateral or bilateral renal agenesis renal hypodysplasia, VUR | branchio-oto-renal (BOR) syndrome |
| 7 | *FGF20* | fibroblast growth factor 20 | 3 | 1688 | Autosomal Recessive | Bilateral renal agenesis | - |
| 8 | *FRAS1* | Fraser Extracellular Matrix Complex Subunit 1 | 74 | 15624 | Autosomal Dominant/Recessive | Renal agenesis/hypodysplasia | Ear and heart defects, syndactyly cryptophthalmos |
| 9 | *FREM2* | FRAS1 related extracellular matrix protein 2 | 24 | 16070 | Autosomal Dominant/Recessive | Renal agenesis/hypodysplasia | Ear and heart defects, syndactyly cryptophthalmos |
| 10 | *GATA3* | GATA binding protein 3 | 6 | 3078 | Autosomal Dominant | Renal dysplasia | Hypoparathyroidism, heart defects, immune deficiency, deafness |
| 11 | *HNF1B* | hepatocyte nuclear factor-1β | 9 | 2977 | Autosomal Dominant | Renal hypodysplasia, cysts | Diabetes |
| 12 | *PAX2* | paired box 2 | 11 | 4140 | Autosomal Dominant | Vesicoureteral reflux, renal hypoplasia | Optic nerve colobomas, hearing loss |
| 13 | *REN* | renin | 10 | 1447 | Autosomal Recessive | Absence or incomplete differentiation of proximal tubules | Pulmonary hypoplasia (Potter sequence), skull abnormalities |
| 14 | *RET* | ret proto-oncogene | 20 | 5659 | Autosomal Dominant | Renal agenesis | multiple endocrine neoplasia, familial medullary thyroid carcinoma |
| 15 | *ROBO2* | roundabout guidance receptor 2 | 26 | 8946 | Autosomal Dominant | VUR, ureterovesical junction defects | Cholangiocarcinoma |
| 16 | *SALL1* | spalt-like transcription factor 1 | 3 | 5253 | Autosomal Dominant | Renal hypodysplasia, renal agenesis | Limb, ear, anal abnormalities |
| 17 | *SIX1* | SIX homeobox 1 | 2 | 3996 | Autosomal Dominant | Renal hypodysplasia, VUR | Deafness, ear defects, branchial cysts |
| 18 | *SIX2* | SIX homeobox 2 | 2 | 2205 | Autosomal Dominant | Renal hypodysplasia | - |
| 19 | *SIX5* | SIX homeobox 5 | 3 | 3318 | Autosomal Dominant | Renal hypodysplasia, VUR | Deafness, ear defects, branchial cysts |
| 20 | *SOX17* | SRY-box 17 | 2 | 2342 | Autosomal Dominant | VUR, UPJO | Congenital heart diseaese |
| 21 | *UMOD* | uromodulin | 11 | 2477 | Autosomal Dominant | Medullary cystic kidney disease type 2 | Hyperuricemia |
| 22 | *UPK3A* | uroplakin 3A | 6 | 1051 | Autosomal Dominant | Renal adysplasia | Subtle facial and limb defects |
| 23 | *WT1* | Wilms tumor 1 | 10 | 3122 | Autosomal Dominant | Kidney malformations | Gentital malformations |
| 24 | *BMP7* | bone morphogenetic protein 7 | 7 | 4013 | Autosomal Dominant | Urethral malformations | Coloboma |
| 25 | *DSTYK* | dual serine/threonine and tyrosine protein kinase | 13 | 7874 | Autosomal Dominant | Renal hypodysplasia, UPJO | Epilepsy in 2 out of 7 affected |
| 26 | *FOXC1* | forkhead box C1 | 1 | 3926 | Autosomal Dominant | Ectopic budding, Hypoplasia | Axenfeld-Rieger syndrome |
| 27 | *GDNF* | glial cell derived neurotrophic factor | 3 | 3810 | Autosomal Dominant | Urinary tract malformations, Ectopic kidney, VUR, Unilateral Agenesis | Hirschsprung disease, Tourette syndrome |
| 28 | *MUC1* | mucin 1, cell surface associated | 8 | 1836 | Autosomal Dominant | Medullary cystic kidney disease type 1 | - |
| 29 | *TRAP1* | TNF receptor-associated protein 1 | 18 | 2296 | Autosomal Recessive | VUR, renal agenesis | VACTERL association |
| 30 | *WNT4* | wingless-type MMTV integration site family member 4 | 5 | 3845 | Autosomal Dominant | Renal hypodysplasia | Female-to-male sex reversal, adrenal dysplasia, lung dysplasia (SERKAL) |
| 31 | *TNXB* | tenascin XB | 13 | 3125 | Autosomal Dominant | VUR | Joint hypermobility |
